# Dietary and environmental factors affecting the dynamics of the gut bacteria in Tibetan Awang sheep (*Ovis aries*) across divergent breeding models

**DOI:** 10.3389/fmicb.2025.1502898

**Published:** 2025-02-05

**Authors:** Yuhao Wang, Xinping Wang, Yirong Wang, Songyu Liao, Zhaxi Pubu, Jiangcuo Silang, Lixu Chai, Siyue Zhao

**Affiliations:** ^1^College of Water Conservancy and Hydropower, Sichuan Agricultural University, Ya'an, Sichuan, China; ^2^Xizang Changdu Animal Husbandry Station, Changdu, Xizang, China; ^3^College of Life Science, Sichuan Agricultural University, Ya'an, Sichuan, China

**Keywords:** Awang sheep, Tibet, gut microbiota, 16S rRNA high-throughput sequencing, bacterial composition

## Abstract

**Introduction:**

Tibetan Awang sheep (*Ovis aries*), indigenous to the Qinghai-Tibet Plateau, are highly adapted to high-altitude environment. However, knowledge regarding their gut bacterial composition remains limited.

**Methods:**

A comprehensive 16S rRNA highthroughput sequencing was performed on fecal samples from 15 Awang sheep under pure grazing, semi-captivity, and full captivity breeding models.

**Results:**

Our results revealed that Firmicutes and Bacteroidetes were the most abundant bacterial phyla, while *Christensenellaceae_R-7_group*, *Romboutsia*, *Rikenellaceae_RC9_gut_group*, *Ruminococcus*, and *Bacteroides* were prevalent genera in the gut microbiota of Awang sheep. Meanwhile, the predominant presence of *Bacteroides* with increasing altitude of breeding locations indirectly demonstrates its crucial role in mediating energy acquisition among Awang sheep at high altitudes. Furthermore, PCoA and ANOSIM analysis exhibited significant differences in bacterial composition across all breeding models (*r* > 0.6, *p* < 0.001). *Christensenellaceae_R-7_group*, *Romboutsia*, and *Ruminococcus* were significantly abundant in the pure grazing breeding model, while *Rikenellaceae_RC9_gut_group* and *Bacteroides* were more abundant in the semi-captivity breeding model. An abnormally high abundance of *Acinetobacter* indicated a potential risk of *Acinetobacter* infection in the fully captive group. The environmental association analysis exhibited that meadows diet (*R*^2^ = 0.938, Pr[>r] = 0.001) and altitude (*R*^2^ = 0.892, Pr[>r] = 0.001) had significant effects on the dominant genera, explaining a substantial proportion of the total variation in community composition.

**Discussion:**

Our study indicated that breeding conditions significantly impact the gut microbiota of Awang sheep. The environmental association analysis underscores the importance of diet and altitude in shaping the gut microbiota of Awang sheep. The present findings provide insights into the microbiota dynamics of Awang sheep and offer guidance for their scientific husbandry management.

## Highlights

- Microbiota Composition and Breeding Models: The gut microbiota of Tibetan Awang sheep varies significantly across pure grazing, semi-captivity, and full captivity models, with Firmicutes and Bacteroidetes as the most abundant phyla.
- Environmental and Dietary Influences: Meadow’s diet and altitude significantly impact the dominant genera in Awang sheep, explaining a substantial proportion of the total variation in community composition.
- Functional Predictions: PICRUSt predictions indicate potential functional differences among the microbiota, particularly in metabolic pathways, signal transduction, and transport systems, across different breeding models.

## 1 Introduction

The Tibetan Awang sheep (*Ovis aries*), a treasured breed native to the Qinghai-Tibet Plateau, thrives primarily in Gongjue County and the surrounding regions of Changdu city (Wang and Yang, 2016). This breed has evolved to adapt to the high-altitude environment, characterized by low temperatures, low oxygen pressure, and limited growing seasons for forage (Liang et al., 2023). The Awang sheep exhibit exceptional resilience, serving as a pure source of nutrition in alpine grasslands (Song et al., 2016). However, their adaptation to captive breeding conditions is compromised due to higher incidence of diseases (Liu L. et al., 2022).

Gut microbiota plays a crucial role in ruminant health by significantly influencing immune system development and function, nutrient absorption, and metabolism (Zhang Y. et al., 2021). As a branch of ruminants, Awang sheep exhibit a complex interplay between their gut microbiota and digestive physiology, overall well-being, as well as their susceptibility to various diseases. Recent genetic and phylogenetic investigations have underscored the unique position and vulnerabilities of Awang sheep, particularly their low genetic diversity stemming from a single haplogroup (A), which is predominantly observed in Asian sheep populations (Liu et al., 2020). This low genetic diversity may exacerbate extinction risks and the fragility of their gut microbiota ecosystem. The gut microbiota has evolved closely with the host, becoming an integral component of the organism. In a healthy state, the gut microbiota and host coexist harmoniously, maintaining microecological balance and organ health. However, environmental changes, particularly in breeding methods influenced by ecological, environmental, and anthropogenic factors (especially diet structure), can disrupt this balance, leading to disease development (Zhao et al., 2021).

Understanding the gut microbiota’s role in Awang sheep’s adaptability and health is essential for improving their production performance under different breeding models (Wang et al., 2023; Wanjala et al., 2023). Despite extensive research on the gut microbiota of Tibetan sheep in Qinghai, Yunnan, and Gansu provinces (Lv et al., 2021; Wang et al., 2022), studies on the gut microbiota structure of Tibetan Awang sheep are lacking. The effects of different feeding methods on their gut microbiota and associated environmental and dietary adaptability remain unclear. Therefore, there is an urgent need to conduct studies on the gut microbiota structure specific to Awang sheep.

Our project aims to study the gut microbiota dynamics of Awang sheep and their consequences for animal husbandry and science. In this study, 16S rRNA high-throughput sequencing was employed to elucidate the disparities in gut microbiota communities of Awang sheep across various breeding models, encompassing pure grazing, semi-captivity, and full captivity. This research will provide insights into the adaptive evolutionary mechanisms of Awang sheep during the transition from grazing to full captivity, from a microbial perspective, and offer guidance for their scientific husbandry management.

## 2 Methods

### 2.1 Sample collection

Fecal samplings were conducted on June 5^th^, 2023, with the assistance and approval of the Xizang Changdu Animal Husbandry Station, China, from three bases: Gongjue County (30°54’N, 98°52′E), Changdu Jueyong Breeding Farm (31°34’N, 97°93′E), and Gongjue Zangdong Biotechnology Co., Ltd. (30°89’N, 98°26′E). A total of 15 fecal samples were collected from healthy Awang sheep directly after defecation with sterilized 50 mL tube. The samples were snap-frozen using liquid nitrogen, and transported to the laboratory on dry ice. The samples were categorized into three breeding models: pure grazing (aw_fm), semi-captivity (aw_bs), and full captivity (aw_qs), each consisting of five male sheep aged 2 years. Pure grazing sheep weighed 25–30 kg and were fed on meadows, semi-captivity sheep weighed 28–33 kg and were fed meadows, hay, and Genkwa root, and full captivity sheep weighed 27–33 kg and were fed concentrates. All sheep were in a healthy condition. More details are summarized in Table 1.

**Table 1:** Details of sample collection.

Breeding model	Sample ID	gender	Age (year)	Weight (kg)	Dietary
Pure grazing (aw_fm)	aw_fm_01	♂	2	25	Meadows
	aw_fm_02	♂	2	29	Meadows
	aw_fm_03	♂	2	28	Meadows
	aw_fm_04	♂	2	30	Meadows
	aw_fm_05	♂	2	28	Meadows
Semi-captivity (aw_bs)	aw_bs_01	♂	2	30	Meadows, hay, Genkwa root
	aw_bs_02	♂	2	28	Meadows, hay, Genkwa root
	aw_bs_03	♂	2	32	Meadows, hay, Genkwa root
	aw_bs_04	♂	2	28	Meadows, hay, Genkwa root
	aw_bs_05	♂	2	33	Meadows, hay, Genkwa root
Full captivity (aw_qs)	aw_qs_01	♂	2	33	Concentrates
	aw_qs_02	♂	2	31	Concentrates
	aw_qs_03	♂	2	27	Concentrates
	aw_qs_04	♂	2	29	Concentrates
	aw_qs_05	♂	2	32	Concentrates
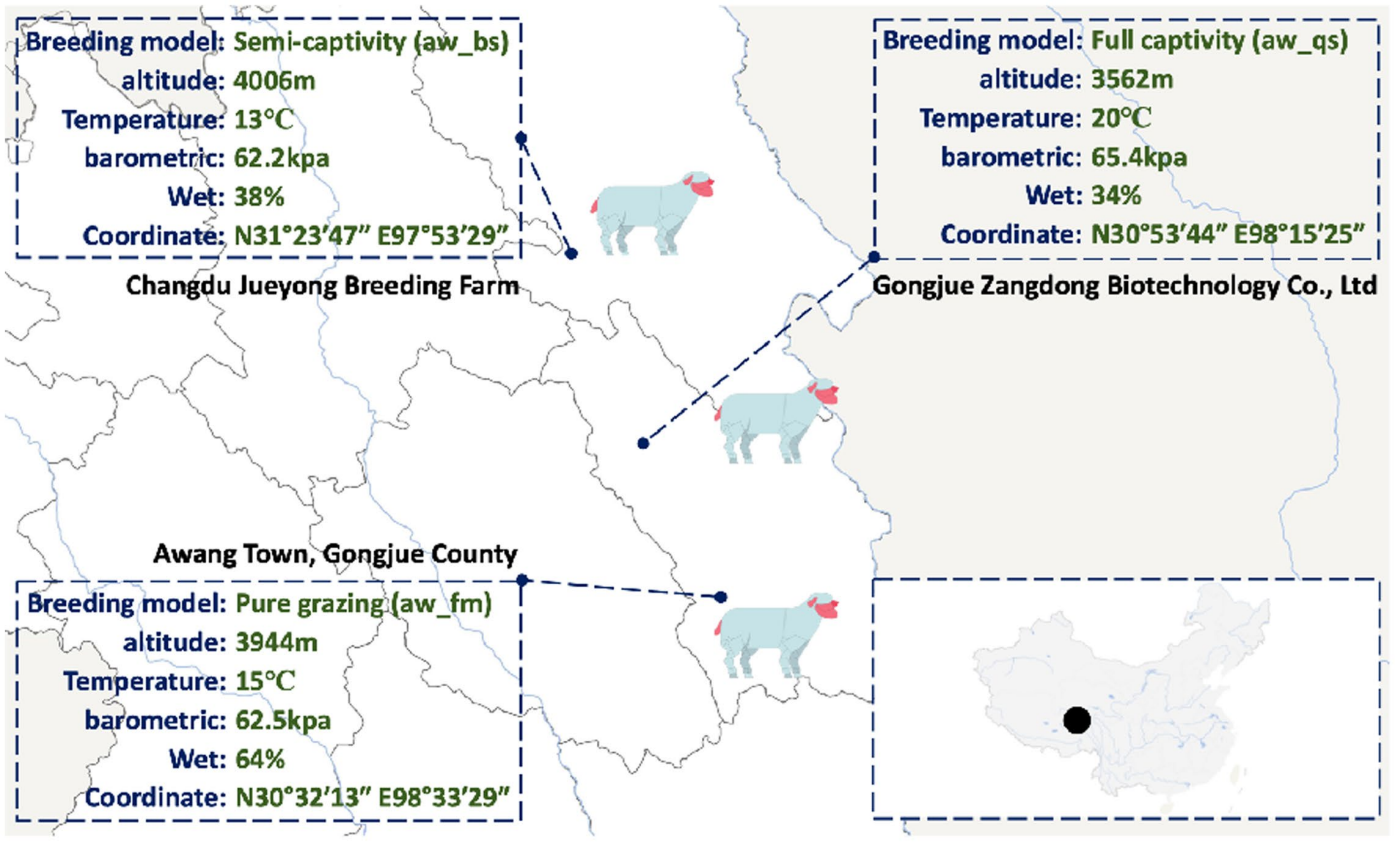					

Samples starting with ‘aw_fm’ come from Awang Town, Gongjue County (pure grazing), ‘aw_bs’ from Changdu Jueyong Breeding Farm (semi-captivity), and ‘aw_qs’ from Gongjue County Zangdong Biotechnology Co., Ltd. (full captivity). ♂ stands for the male. Age range: 0–1 years old for juvenile sheep, 2–8 years for adult sheep.

### 2.2 DNA extraction, library construction, and Illumina sequencing

The total genomic DNA from the samples was extracted using the QIAamp^®^ PowerFecal^®^ Pro DNA Kit (Qiagen Inc., German) according to the manufacturer’s instructions. DNA concentration and purity were monitored on 1% agarose gel. The hypervariable region V4 (83bp) of the 16S rRNA genes were amplified using primers 520F (5′- barcode + AYTGGGYDTAAAGNG-3) and 802R (5’-TACNVGGGTATCTAATCC-3′). The amplicon quality was visualized using gel electrophoresis. The PCR products were purified with Agencourt AMPure XP beads (Beckman Coulter, United States) and quantified using a Qubit dsDNA assay kit. Sequencing was performed on an Illumina NovaSeq6000 platform with two paired-end read cycles of 250 bases each (Illumina Inc., San Diego, CA; sequencing service provided by OE Biotech Company, Shanghai, China).

### 2.3 Bioinformatic analysis

After sequencing, the raw data were obtained in FASTQ format. Paired-end reads were then preprocessed using Cutadapt^1^ to detect and trim off the adapter sequences. After trimming, the paired-end reads were filtered for low-quality sequences, denoised, merged, and chimeras were detected and removed using DADA2,^2^ a tool for high-resolution amplicon sequence variant analysis, with the default parameters of QIIME2 (Version 1.7.0, http://qiime.org/index.html), a microbiome analysis platform. A similarity threshold of 100% is set, and sequences with similarity exceeding this threshold are grouped into a single ASV. Finally, the software outputs the representative reads and the ASV abundance table. The representative read for each ASV was selected using the QIIME2 package. All representative reads were annotated and blasted against the Silva database Version 138 (for 16S rDNA) using q2-feature-classifier^3^ with the default parameters. Functional predictions were employed by PICRUSt2.^4^

### 2.4 Statistical analysis and data visualization

Most of the subsequent statistical analysis was performed using Microsoft Excel (Microsoft Inc.), Python^5^ and R (package: ggplot2 and vegan).^6^ The cladogram with circular representations of taxonomic and phylogenetic trees was generated using GraPhlAn2.^7^ Indices of rumen bacterial richness (Chao1 index) and diversity (Shannon index) were calculated using software R (see text footnote 6) for alpha diversity analysis. The ANOVA (Analysis of Variance) method was employed to test for statistical differences among groups. For beta diversity, the principal coordinate analysis (PCoA) was performed based on the ASV-based Weighted Unifrac and Bray-Curtis distance matrices using R software (see text footnote 6) with the GUniFrac, ape and ggplot2 packages (Jiang et al., 2013). A one-way analysis of similarity (ANOSIM) (Li et al., 2017) was conducted to assess the differences in beta diversity among all the breeding model of Awang sheep. The specific species that had significant differences at each level were identified and visualized through LDA effect size (LEfSe) analysis performed online.^8^ Canoco5^9^ was employed to conduct Redundancy Analysis (RDA). Mantel test was performed using R with dplyr, linkET and ggplot2 packages. Cytoscape (Version 3.6.1, http://cytoscape.org) was used to visualize the network displaying correlations between different genera, correlation coefficients were calculated using the Spearman method with RStudio.

## 3 Results

### 3.1 Overview of sequencing

In our sequencing, we successfully generated 871,682 high-quality reads. These reads were further classified into 4,141 distinct amplicon sequence variants (ASVs). The high density of the sequencing data, with 23.9% of the values being non-zero, reflects the richness and complexity of the gut microbiota community in the Awang sheep samples. A summary of the read counts per sample reveals that the minimum was 47,290 reads, the maximum was 63,605 reads, the median was 58,964 reads, and the mean was 58,112.133 reads, with a standard deviation of 3,636.515 reads. Meanwhile, the detailed read counts for each sample range from 47,290 to 63,605, further demonstrating the consistency in sequencing depth across the samples. The detailed sequencing data is shown in Supplementary Table S1. Besides, the rarefaction curves (Supplementary Figure S1) gradually became flat and reached a plateau with more data indicating that the number of ASVs for each sample was sufficient and reasonable. The rank abundance curves that reflected the evenness and abundance of species in fecal samples horizontally and vertically were demonstrated in Supplementary Figure S2.

**Figure 1:**
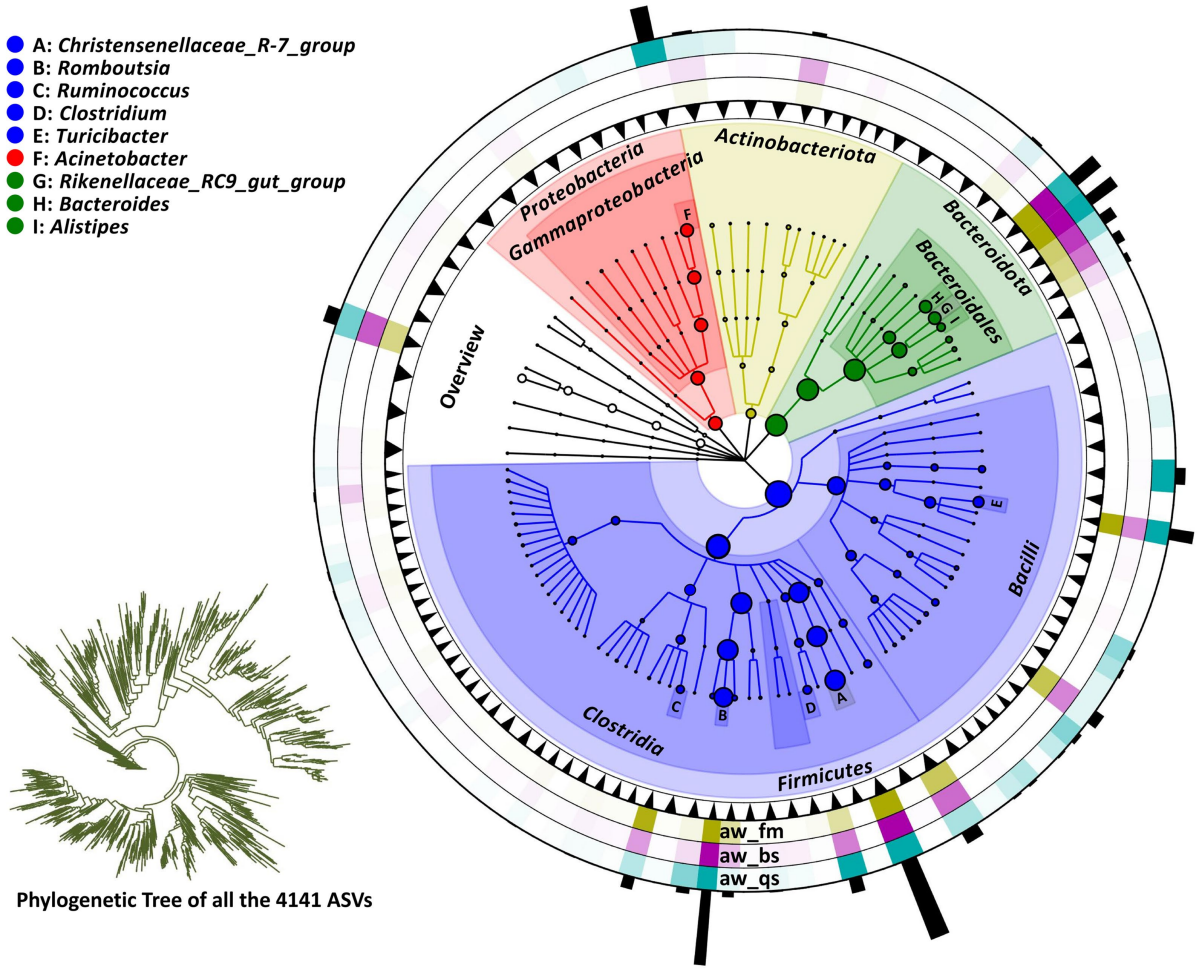
Overview phylogenetic circle plot of dominant taxa of Awang sheep and ASVs phylogenetic tree. The overview phylogenetic circle plot illustrates the taxonomic relationships and diversity structure among the dominant microbial communities, with the outer rings representing the higher taxonomic levels and the inner rings indicating the lower levels. Taxa with an average relative abundance higher than 1% are included in the plot, providing a clear visualization of the dominant microbial communities and their evolutionary relationships. The taxonomic level ranges from phylum to genus from inner to outer circles. The diameter of nodes indicates the abundance at different taxonomic levels, and different colors denote different taxonomic clades. aw_fm, aw_bs, and aw_qs represents pure grazing, semi-captivity, and full captivity breeding models, respectively.

Moreover, these 4,141 ASVs were mainly classified into 20 phyla, 37 classes, 99 orders, 165 families and 309 genera. Meanwhile, an overview phylogenetic tree of the taxa, of which average relative abundance higher than 1%, was generated by Graphlan.py and Graphlan_annot.py script to better exhibit the taxonomic relationships and diversity structure among the dominant microbial communities (Figure 1). It is obvious that Firmicutes, Bacteroidetes, Proteobacteria, and Actinobacteriota were the dominant phyla, while *Christensenellaceae*_*R-7_group*, *Romboutsia*, *Ruminococcus*, *Clostridium*, *Turicibacter*, *Acinetobacter*, *Rikenellaceae*_*RC9_gut_group*, and *Bacteroides* were the dominant genera in the gut microbiota of Awang sheep.

### 3.2 Microbiome diversity analysis of Awang sheep breeding models reveals distinct clustering patterns

**Figure 2:**
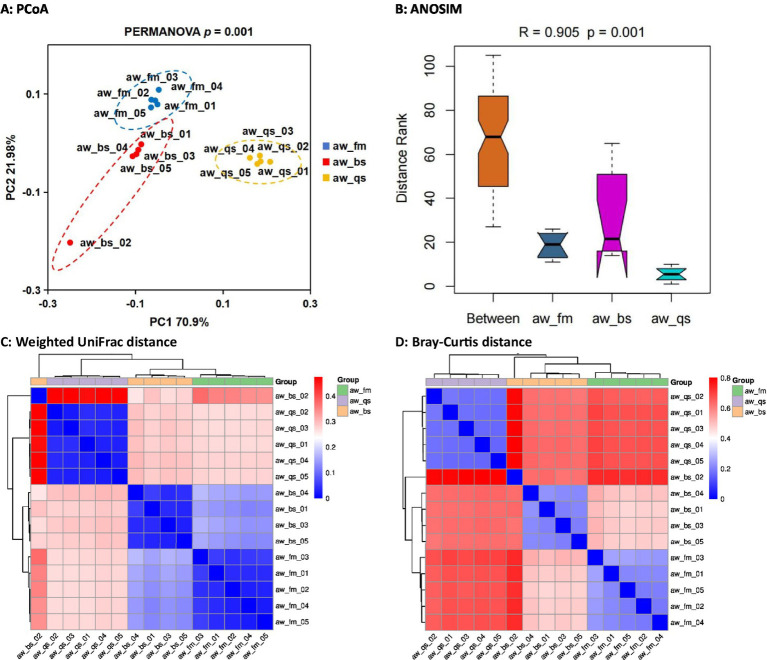
Comprehensive analysis of gut microbiota diversity in Awang sheep under different breeding models. This figure comprehensively analyzes gut microbiome diversity in Awang sheep across pure grazing, semi-captivity, and full captivity breeding models. It includes four key components: **(A)** Principal Coordinates Analysis (PCoA) plot showing distinct clustering patterns among the breeding models, **(B)** ANOSIM plot verifying significant differences in gut microbiota profiles, **(C)** hierarchical clustering heatmap based on Weighted UniFrac distance matrix, and **(D)** hierarchical clustering heatmap based on Bray-Curtis distance matrix. These four visualizations demonstrate the diversity discrepancy among the breeding models. Note: In the heatmap, the bluer the color is, the closer the distance between samples is, and the higher the similarity is. aw_fm, aw_bs, and aw_qs represents pure grazing, semi-captivity, and full captivity breeding models, respectively.

Initially, the diversity discrepancy of the gut microbiota of Awang sheep among the pure grazing, semi-captivity and full captivity breeding model was calculated based on Weighted UniFrac and Bray-Curtis distance matrices. The PCoA (Principal Coordinates Analysis) plots showed that each group tended to cluster within the respective group (Figure 2A). Each point in the plot represents a sample, and samples of the same color belong to the same group. Samples within the same group are close in distance and distinct from other groups, indicating a good clustering effect. This PCoA result was subsequently verified by ANOSIM, which indicated significant differences in the gut microbiota profiles among the breeding models (*r* > 0.6, *p* < 0.001) (Figure 2B). Based on the distance matrices obtained from the Weighted UniFrac and Bray-Curtis distance algorithms, the heatmap of hierarchical clustering visualized the proximity of our sample branches (Figures 2C,D).

**Figure 3:**
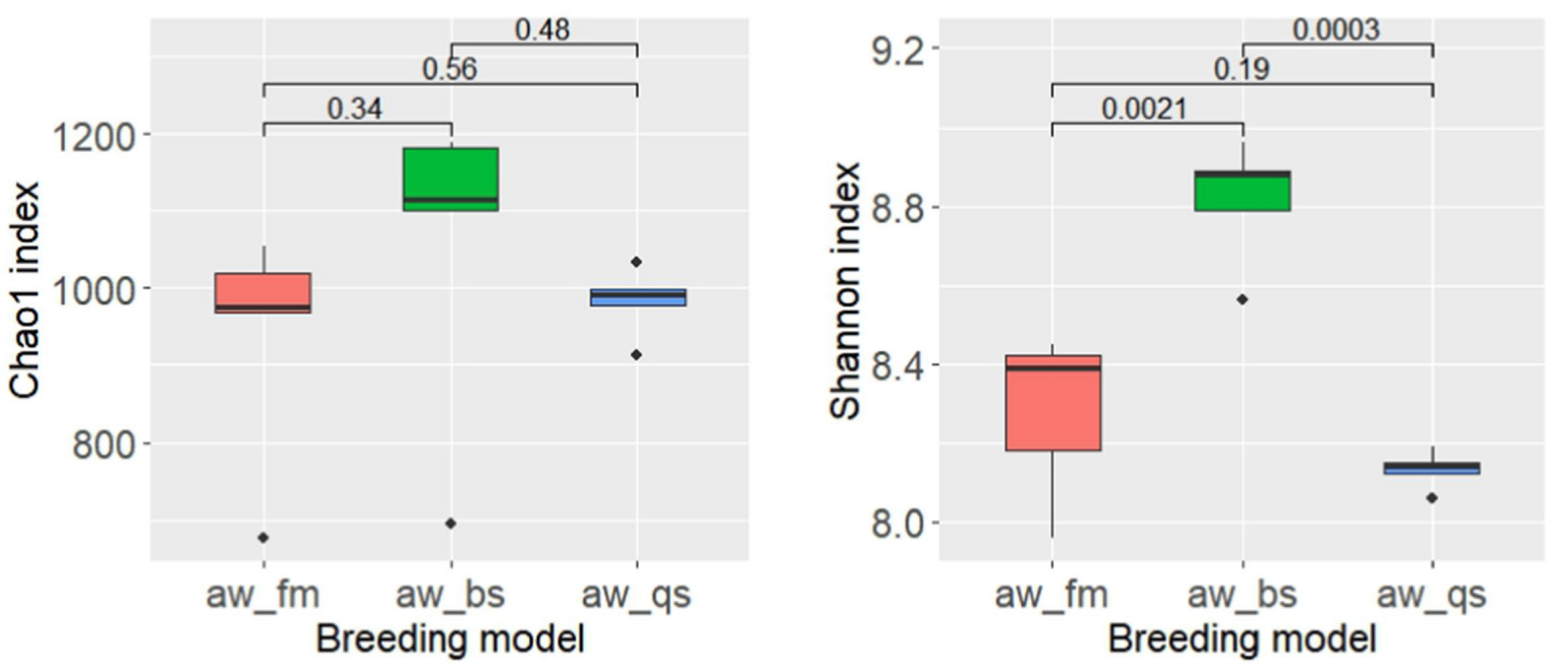
Boxplot of *α* diversity index (Chao1 and Shannon) of gut microbiota across all breeding models. aw_fm, aw_bs, and aw_qs represents pure grazing, semi-captivity, and full captivity breeding models, respectively.

Subsequently, we compared the alpha diversity of Awang sheep gut microbiota across the three breeding models: pure grazing (aw_fm), semi-captivity (aw_bs), and full captivity (aw_qs), using Chao1 (richness) and Shannon (evenness) indices (Figure 3). Specifically, the Chao1 index was 938.6 ± 150.2, 1055.3 ± 205.9, and 982.8 ± 43.9, respectively, for the pure grazing, semi-captivity, and full captivity models. Similarly, the Shannon index was 8.28 ± 0.21, 8.82 ± 0.15, and 8.13 ± 0.05, respectively. ANOVA analysis revealed no significant difference (*p* > 0.05) in the Chao1 index among the breeding models. However, for the Shannon index, a significant difference was observed between the pure grazing (aw_fm) and semi-captivity (aw_bs) models (*p* < 0.01), as well as between the semi-captivity (aw_bs) and full captivity (aw_qs) models (*p* < 0.001).

### 3.3 Bacterial composition at phylum and genus level

**Figure 4:**
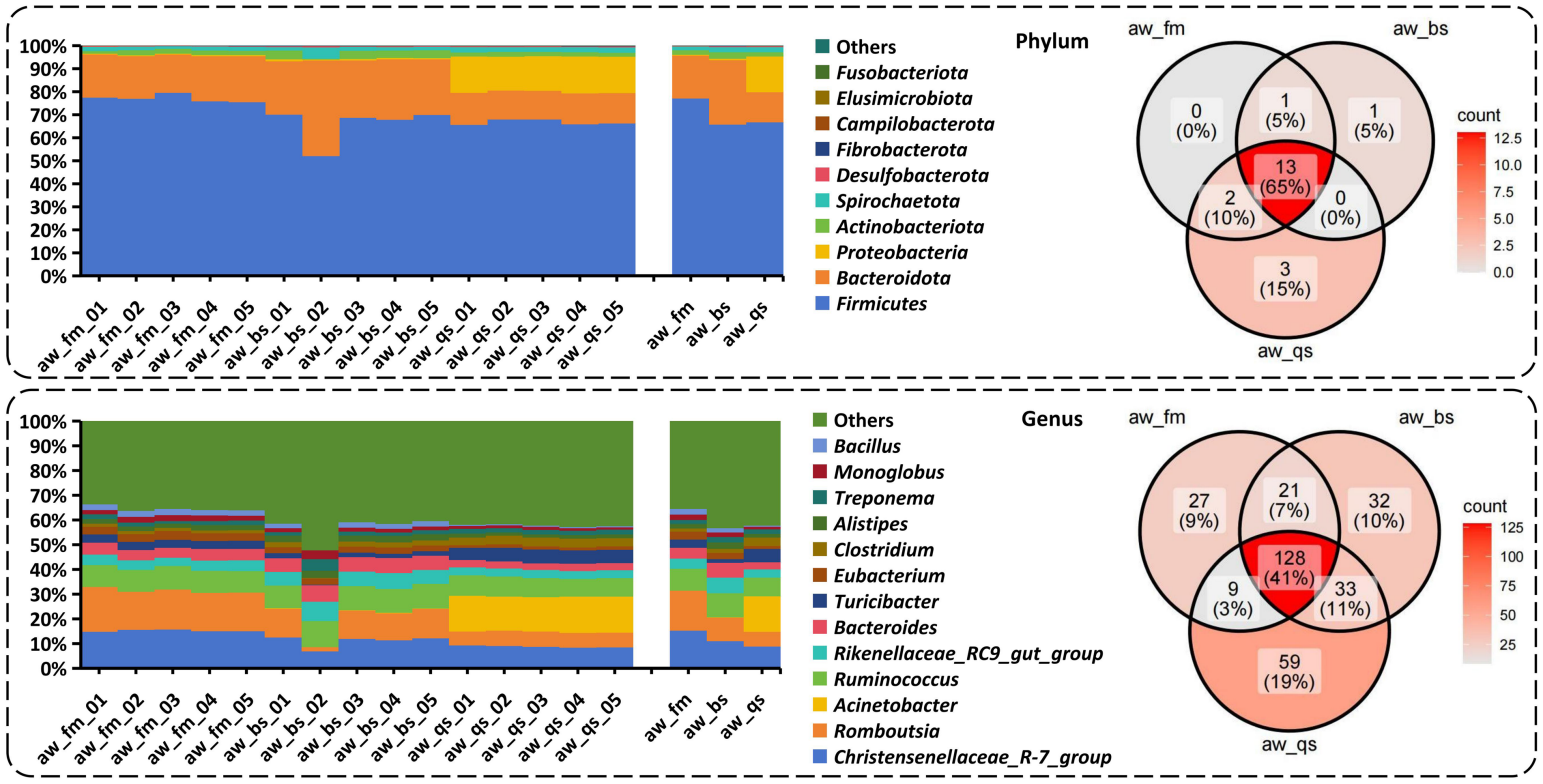
Composition and distribution of gut microbiota phyla and genera across different breeding model by percentage stacked histogram of relative abundance and Venn diagram. aw_fm, aw_bs, and aw_qs represents pure grazing, semi-captivity, and full captivity breeding models, respectively.

Based on a comprehensive analysis of gut microbiota across different breeding models, we present a percentage stacked histogram of relative abundance and a Venn diagram to illustrate the composition and distribution of gut microbiota phyla and genera (Figure 4).

In the pure grazing model, 17 phyla, 26 classes, 63 orders, 105 families, 187 genera, and 261 species were detected from the gut of Awang sheep. Firmicutes was the most abundant phylum, accounting for 77.06% ± 1.59%, followed by Bacteroidetes (18.65% ± 1.27%) and Proteobacteria (0.42% ± 0.07%) (Figure 4). Meanwhile, *Romboutsia* (16.19% ± 1.17%) was the predominant genus followed by *Christensenellaceae_R-7_group* (15.23% ± 0.42%), *Ruminococcus* (11.93% ± 0.28%), and *Bacteroides* (4.41% ± 0.34%).

In the semi-captivity model, 16 phyla, 27 classes, 71 orders, 117 families, 215 genera, and 322 species were detected from the gut of Awang sheep. Firmicutes (65.68% ± 7.69%) was the most abundant phylum, followed by Bacteroidetes (28.05% ± 7.66%) and Proteobacteria (0.56% ± 0.20%) (Figure 4). Meanwhile, *Christensenellaceae_R-7_group* (10.97% ± 2.36%) was the predominant genus followed by *Ruminococcus* (9.81% ± 0.49%), *Romboutsia* (9.54% ± 4.33%), and *Bacteroides* (5.96% ± 0.42%).

In the full captivity model, 19 phyla, 30 classes, 83 orders, 134 families, 230 genera, and 355 species were detected from the gut of Awang sheep. Firmicutes (66.72% ± 1.15%) was the most abundant phylum followed by Proteobacteria (15.52% ± 0.57%) and Bacteroidetes (13.09% ± 0.64%) (Figure 4). Meanwhile, *Acinetobacter* (14.32% ± 0.37%) was the predominant genus followed by *Christensenellaceae_R-7_group* (8.80% ± 0.42%), *Ruminococcus* (7.69% ± 0.47%), *Romboutsia* (5.95% ± 0.24%), and *Bacteroides* (2.86% ± 0.13%).

Based on the comprehensive analysis of the Venn diagram (Figure 4), it is evident that 13 phyla, including Firmicutes, Bacteroidetes, Proteobacteria, Actinobacteriota, Spirochaetota, Desulfobacterota and Fibrobacterota, as well as 128 genera such as *Christensenellaceae*_*R-7_group*, *Romboutsia*, *Acinetobacter*, *Rikenellaceae*_*RC9_gut_group*, and *Bacteroides*, were shared by all the breeding models. The shared phyla and genera above were abundant in our sequencing.

Alternatively, 1 unique phylum Marinimicrobia_(SAR406_clade) was identified in the semi-captivity breeding model, while 3 unique phyla, Nitrospirota, Bdellovibrionota, and NB1-j, were identified in the full captivity breeding model, respectively. Moreover, 27 unique genera, including *Paludicola*, *Mycobacterium*, and others, were identified in the pure grazing breeding model. In the semi-captivity breeding model, 32 unique genera were identified, such as *Kandleria*, *Pseudoramibacter*, and others. Besides, 59 unique genera, comprising *Planococcus*, *Carnobacterium*, and others, were identified in the full captivity breeding model. However, none of the unique phyla and genera were prevalent in accordance with previous relative abundance results.

### 3.4 Microbial population difference

**Figure 5:**
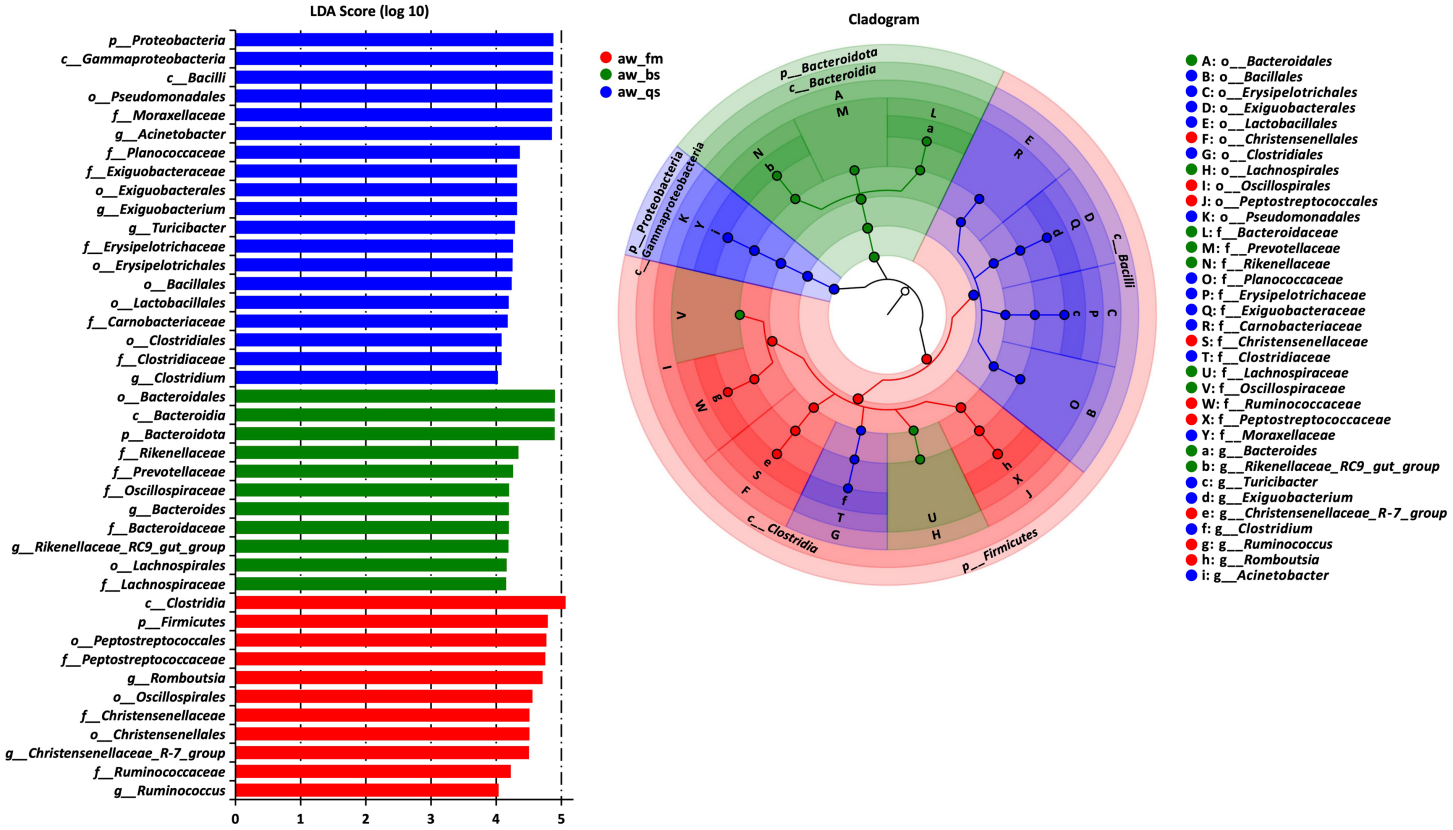
The results of LEfSe (LDA Effect Size) analysis. The histogram of the LDA score showed the biomarkers with statistical differences among the groups. The influencing degree of species was expressed by bar length in the histogram. In the cladogram, the circle radiated inside-out demonstrated the classification (from phylum to genus). Each small circle at a different classification represented a taxon and the circle is proportional to the contribution (LDA score) of the specific taxon in certain groups. Red, green and blue dots represent the core bacterial populations in the respective breeding model. aw_fm, aw_bs, and aw_qs represents pure grazing, semi-captivity, and full captivity breeding models, respectively.

To identify significant taxa (LDA score > 4, *p* < 0.001), an LDA effect size (LEfSe) analysis was conducted across all breeding models, the cladogram clearly illustrated the core taxa that exhibited remarkable differences across all levels and breeding models (Figure 5).

In the pure grazing model (aw_fm), 11 taxa displayed significant discrepancies in relative abundance. These comprised the phylum Firmicutes, the class *Clostridia*, orders including *Peptostreptococcales*, *Oscillospirales* and *Christensenellales*, several families including *Peptostreptococcaceae*, *Christensenellaceae* and *Ruminococcaceae*, and various genera, such as *Romboutsia*, *Christensenellaceae*_*R-7_group*, and *Ruminococcus*.

A similar analysis in the semi-captivity model (aw_bs) revealed 11 significant taxa. These were dominated by the phylum Bacteroidetes, including class *Bacteroidia*, order *Bacteroidales*, order *Lachnospirales*, and family *Rikenellaceae*, *Prevotellaceae*, *Oscillospiraceae*, *Bacteroidaceae*, and *Lachnospiraceae*. The genus *Bacteroides* and *Rikenellaceae_RC9_gut_group* also stood out.

In the full captivity model (aw_qs), the LEfSe analysis identified 19 significant taxa. These encompassed the phylum Proteobacteria, with the classes *Gammaproteobacteria* and *Bacilli*, several orders including *Pseudomonadales*, *Exiguobacterales*, *Erysipelotrichales*, *Bacillales*, *Lactobacillales* and *Clostridiales*, and families *Moraxellaceae*, *Planococcaceae*, *Exiguobacteraceae*, *Erysipelotrichaceae*, *Carnobacteriaceae*, and *Clostridiaceae*. Among the genera, *Acinetobacter*, *Exiguobacterium*, *Turicibacter*, and *Clostridium* was particularly notable.

**Figure 6:**
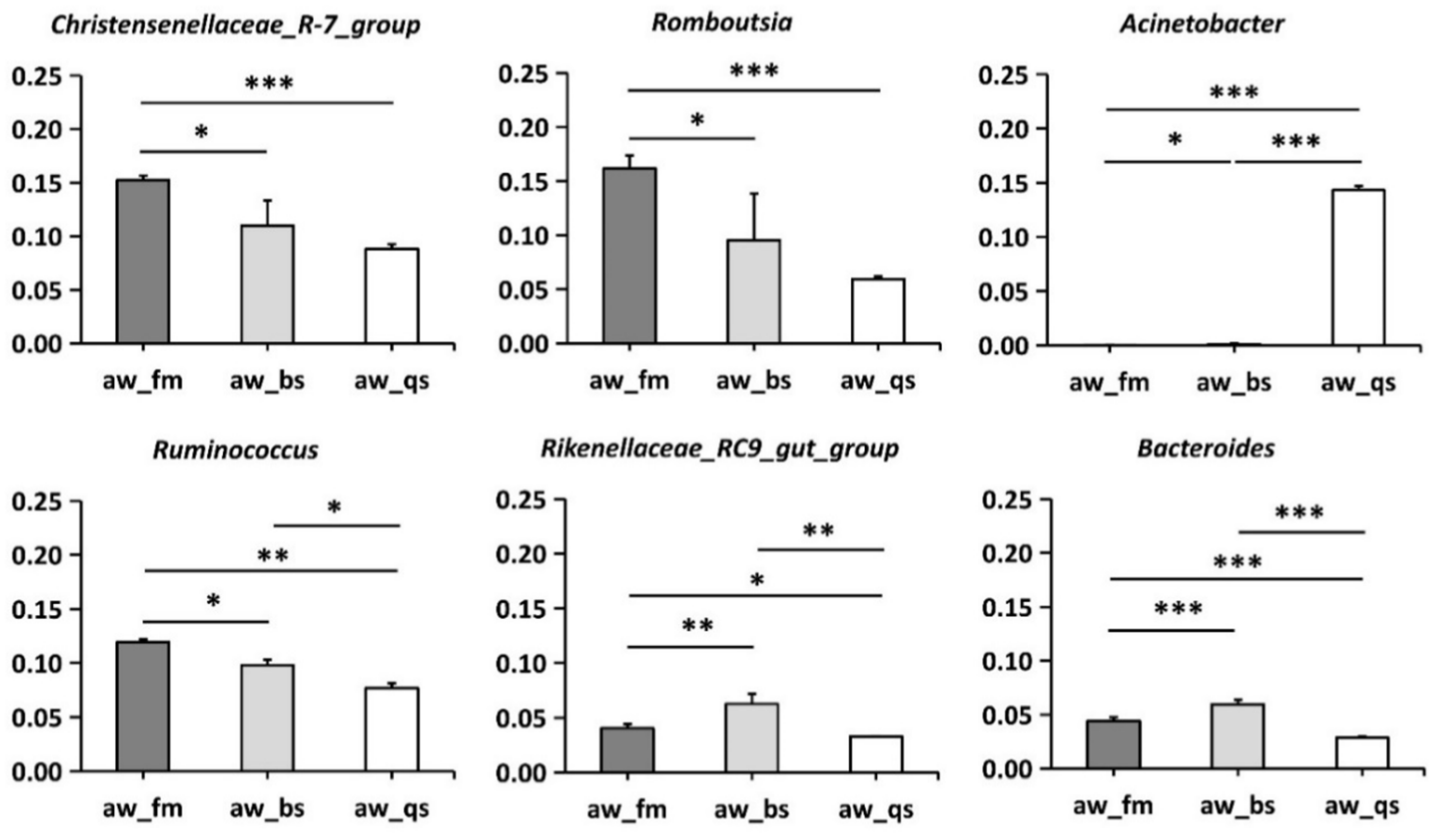
Differential abundance of microbial genera across breeding models in Awang sheep. aw_fm, aw_bs, and aw_qs represents pure grazing, semi-captivity, and full captivity breeding models, respectively. “*” represents *p* < 0.05, “**” represents *p* < 0.01, and “***” represents *p* < 0.001.

To identify differentially abundant microbial genera, including *Christensenellaceae*_*R-7_group*, *Romboutsia*, *Acinetobacter*, *Ruminococcus*, *Rikenellaceae_RC9_gut_group*, and *Bacteroides*, which were prevalent and detected in LEfSe (LDA Effect Size) analysis, we performed deeper statistical tests by applying Kruskal-Wallis across the breeding models. Consistent with the LEfSe analysis, we observed that these six genera exhibited statistically significant differences in abundance (Figure 6). Specifically, *Christensenellaceae*_*R-7_group*, *Romboutsia*, and *Ruminococcus* displayed significantly higher abundance in the pure grazing breeding model compared to the other two breeding models. The gradual decrease in abundance of these three genera is of particular interest, from pure grazing to semi-captive conditions and then to full captivity. Conversely, *Rikenellaceae_RC9_gut_group* and *Bacteroides* were more abundant in the semi-captivity breeding model, while *Acinetobacter* showed significantly increased abundance in the full captivity breeding model. Specifically, *Acinetobacter* exhibited a notably higher abundance in the full captivity breeding model compared to both the pure grazing (*p* < 0.001) and semi-captivity (*p* < 0.001) breeding models.

### 3.5 Correlation among the gut microbiota at the genus level

**Figure 7:**
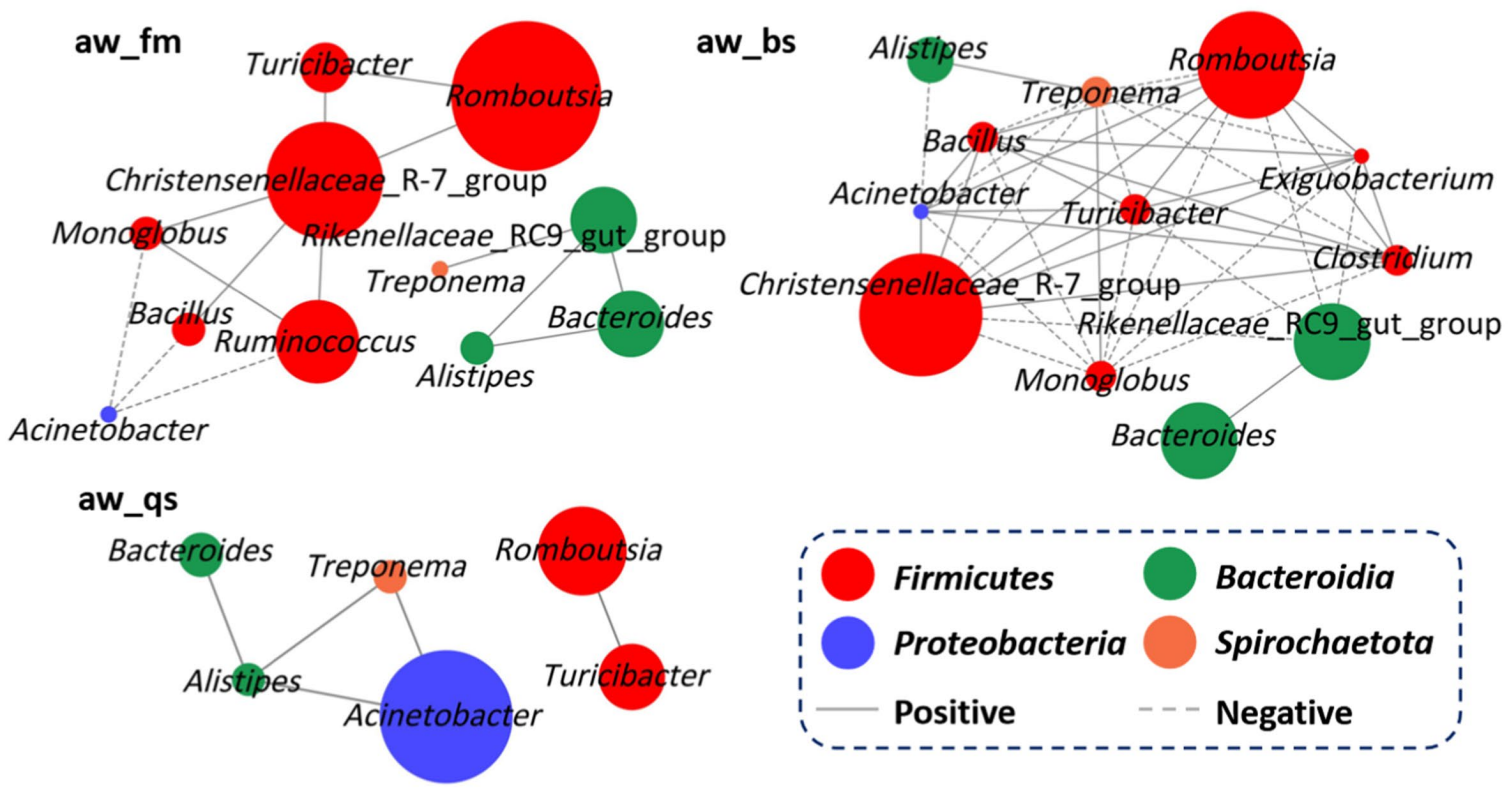
Network analysis depicting potential correlations among genera in different breeding models, with circle size reflecting relative abundance. aw_fm, aw_bs, and aw_qs represents pure grazing, semi-captivity, and full captivity breeding models, respectively.

To uncover the interrelationships of prevalent bacteria, a comprehensive correlation analysis was conducted at the genus level for each breeding model (Figure 7). In the pure grazing model (aw_fm), *Christensenellaceae*_*R-7_group* showed a positive correlation with *Romboutsia* (*R* = 0.91, *p* = 0.031), *Turicibacter* (*R* = 0.97, *p* = 0.006), *Ruminococcus* (*R* = 0.89, *p* = 0.038), *Monoglobus* (*R* = 0.93, *p* = 0.019), and *Bacillus* (*R* = 0.92, *p* = 0.022). *Rikenellaceae*_*RC9_gut_group* displayed a positive correlation with *Bacteroides* (*R* = 0.98, *p* = 0.004), *Alistipes* (*R* = 0.97, *p* = 0.008), and *Treponema* (*R* = 0.93, *p* = 0.021). Separately, *Romboutsia*, *Bacteroides*, and *Ruminococcus* exhibited a positive correlation with *Turicibacter* (*R* = 0.96, *p* = 0.008), *Alistipes* (*R* = 0.98, *p* = 0.003) and *Monoglobus* (*R* = 0.96, *p* = 0.010) respectively. In addition, *Acinetobacter* exhibited a negative correlation with *Ruminococcus* (*R* = −0.92, *p* = 0.025), *Monoglobus* (*R* = −0.92, *p* = 0.028), and *Bacillus* (*R* = −0.90, *p* = 0.035).

In the semi-captivity model (aw_bs), the *Christensenellaceae*_*R-7_group* was positively correlated with *Romboutsia* (*R* = 0.99, *p* = 0.0005), *Acinetobacter* (*R* = 0.93, *p* = 0.021), *Turicibacter* (*R* = 0.99, *p* = 0.0001), *Clostridium* (*R* = 0.98, *p* = 0.002), *Exiguobacterium* (*R* = 0.94, *p* = 0.017), and *Bacillus* (*R* = 0.98, *p* = 0.002). However, it was negatively correlated with the *Rikenellaceae*_*RC9_gut_group* (*R* = −0.93, *p* = 0.021), *Treponema* (*R* = −0.98, *p* = 0.002), and *Monoglobus* (*R* = −0.97, *p* = 0.004). Similarly, *Romboutsia* showed a positive correlation with *Acinetobacter* (*R* = 0.95, *p* = 0.011), *Turicibacter* (*R* = 0.99, *p* = 0.0006), *Clostridium* (*R* = 0.99, *p* = 0.0002), *Exiguobacterium* (*R* = 0.93, *p* = 0.018), and *Bacillus* (*R* = 0.99, *p* = 0.0005), while exhibited a negative correlation with *Rikenellaceae*_*RC9_gut_group* (*R* = −0.89, *p* = 0.042), *Treponema* (*R* = −0.99, *p* = 0.0006), and *Monoglobus* (*R* = −0.98, *p* = 0.001). Furthermore, *Acinetobacter* is positively related to *Turicibacter* (*R* = 0.94, *p* = 0.014), *Clostridium* (*R* = 0.96, *p* = 0.007), and *Bacillus* (*R* = 0.94, *p* = 0.014). Conversely, it was negatively correlated with *Alistipes* (*R* = −0.93, *p* = 0.019), *Treponema* (*R* = −0.97, *p* = 0.005), and *Monoglobus* (*R* = −0.96, *p* = 0.009). Additionally, *Rikenellaceae*_*RC9_gut_group* showed a positive correlation with *Bacteroides* (*R* = 0.96, *p* = 0.009), but negatively correlated with *Turicibacter* (*R* = −0.91, *p* = 0.028) and *Exiguobacterium* (*R* = −0.89, *p* = 0.041). *Turicibacter* was positively correlated with *Clostridium* (*R* = 0.99, *p* = 0.0009), *Exiguobacterium* (*R* = 0.94, *p* = 0.016), and *Bacillus* (*R* = 0.97, *p* = 0.003), while demonstrating a negative correlation with *Treponema* (*R* = −0.99, *p* = 0.001) and *Monoglobus* (*R* = −0.98, *p* = 0.003). *Clostridium* showed a strong positive correlation with *Exiguobacterium* (*R* = 0.95, *p* = 0.014) and *Bacillus* (*R* = 0.97, *p* = 0.002), but a negative correlation with *Treponema* (*R* = −0.99, *p* = 0.00006) and *Monoglobus* (*R* = −0.99, *p* = 0.0002).

In the full captivity model, *Romboutsia* was positively correlated with *Turicibacter* (*R* = 0.97, *p* = 0.005). Meanwhile, *Acinetobacter* showed a positive correlation with *Alistipes* (*R* = 0.89, *p* = 0.037) and *Treponema* (*R* = 0.88, *p* = 0.047). In addition, *Bacteroides* exhibited a strong positive correlation with *Alistipes* (*R* = 0.97, *p* = 0.005). Furthermore, *Alistipes* exhibited a positive correlation with *Treponema* (*R* = 0.92, *p* = 0.022).

### 3.6 Association analysis of environmental factors affecting differences in dominant genera across different breeding models

**Figure 8:**
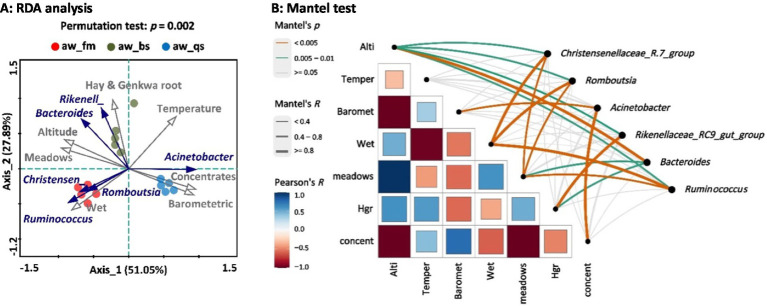
Association analysis of the relationship between environmental factors and dominant genera with significant differences across all the breeding models. **(A)** RDA plot: The points with different colors in the figure represent groups of samples across different breeding models. The grey lines and arrows emanating from the origin represent different environmental factors and genera, respectively. The length of the environmental factor arrows indicates the degree of influence (explained amount) of the environmental factors on the genera. The angle between the environmental factor arrow and the genera arrow represents positive (acute angle) or negative (obtuse angle) correlation, while a right angle indicates no correlation. The vertical distance from the sample point to the extended line representing the environmental factor arrow represents the strength of the influence that an environmental factor has on the sample. The closer the sample point is to the arrow, the stronger the effect of that environmental factor on the sample. Correlation between environmental factors and RDA axes are shown by both length and angle of arrows. Rikenell represents *Rikenellaceae*_*RC9_gut_group*. Christensen represents *Christensenellaceae*_*R-7_group*. Note: aw_fm, aw_bs, and aw_qs represents pure grazing, semi-captivity, and full captivity breeding models, respectively. **(B)** Mantel test plot: Alti represents altitude, Temper represents temperature, Hgr represents hay and genkwa root, and concent represents concentrates.

The application of Redundancy Analysis (RDA) to our data set revealed significant associations between environmental factors and the distribution of microbiota genera in the fecal samples of Awang sheep (Figure 8A). Altitude (*R*^2^ = 0.892, Pr(>r) = 0.001) was the primary environmental factor influencing the change in the dominant genera, followed by Barometric Pressure (*R*^2^ = 0.836, Pr(>r) = 0.001), Wetness (*R*^2^ = 0.772, Pr(>r) = 0.001), and Temperature (*R*^2^ = 0.708, Pr(>r) = 0.003). From a dietary perspective, Meadow (*R*^2^ = 0.938, Pr(>r) = 0.001) diet was the key environmental factor affecting the genera’s change, followed by Concentrates (*R*^2^ = 0.888, Pr(>r) = 0.001) and Hay_and_Genkwa_root (*R*^2^ = 0.733, Pr(>r) = 0.002). Based on RDA and Mantel test, we analyzed the correlation between genera and environmental factors (Figure 8B). Specifically, meadows diet exhibited a moderate correlation with multiple genera, including *Ruminococcus* (*R* = 0.792, *p* = 0.001), *Bacteroides* (*R* = 0.655, *p* = 0.006), *Romboutsia* (*R* = 0.641, *p* = 0.003), and *Christensenellaceae*_*R-7_group* (*R* = 0.639, *p* = 0.003). Hay and genkwa root diet were positively correlated with *Rikenellaceae*_*RC9_gut_group* (*R* = 0.828, *p* = 0.001). Additionally, there was a positive correlation between concentrates (*R* = 0.805, *p* = 0.001), Barometric pressure (*R* = 0.636, *p* = 0.003), and *Acinetobacter*. Furthermore, Wetness was positively correlated with *Ruminococcus* (*R* = 0.862, *p* = 0.002), *Christensenellaceae*_*R-7_group* (*R* = 0.859, *p* = 0.002), and *Romboutsia* (*R* = 0.815, *p* = 0.002). Altitude emerged as an important factor affecting the abundance of *Bacteroides* (*R* = 0.876, *p* = 0.001), with *Romboutsia* (*R* = 0.592, *p* = 0.009) and *Christensenellaceae*_*R-7_group* (*R* = 0.445, *p* = 0.012) also exhibiting some association. Our RDA and Mantel test analysis indicates that meadow diet and altitude are closely related to the changes in these dominant genera and may be important factors driving these changes.

### 3.7 PICRUSt predictions

In this study, we predicted the abundance of COG (Clusters of Orthologous Groups of proteins) and KO (KEGG Orthology) in sequencing. We used PICRUSt2 software to predict the composition of known microbial gene functions, thus statistically analyzing the functional differences among different breeding models.

**Figure 9:**
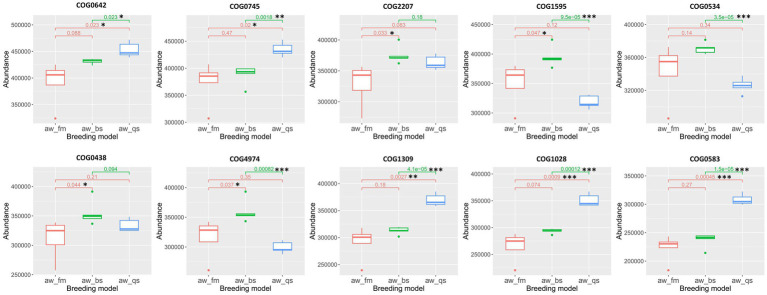
Boxplot representation of COG abundances across different breeding models. The Kruskal-Wallis algorithm was employed to statistically analyze the differences in the predicted COG results among these models. The boxplots illustrate the distribution of abundance values for each COG, with significant differences among the breeding models denoted. aw_fm, aw_bs, and aw_qs represents pure grazing, semi-captivity, and full captivity breeding models, respectively. “*” represents *p* < 0.05, “**” represents *p* < 0.01, and “***” represents *p* < 0.001.

To statistically analyze the predicted COG results across multiple breeding models, we employed the Kruskal-Wallis test and selected the top 10 most enriched COGs, including COG0642 (Signal transduction histidine kinase), COG0745 (DNA-binding response regulator, *OmpR* family, contains REC and winged-helix (wHTH) domain), COG2207 (*AraC*-type DNA-binding domain and *AraC*-containing proteins), COG1595 (DNA-directed RNA polymerase specialized sigma subunit, sigma24 family), COG0534 (Na + −driven multidrug efflux pump), COG0438 (Glycosyltransferase involved in cell wall biosynthesis), COG4974 (Site-specific recombinase *XerD*), COG1309 (DNA-binding transcriptional regulator, *AcrR* family), COG1028 (NAD(P)-dependent dehydrogenase, short-chain alcohol dehydrogenase family), and COG0583 (DNA-binding transcriptional regulator, *LysR* family), for the creation of boxplots (Figure 9). For COG0642, COG0745, COG1309, COG1028, and COG0583, an apparent gradual increase in abundance was observed from pure grazing breeding model to semi-captivity breeding model, and subsequently to full captivity breeding model. Furthermore, these COGs especially COG1309, COG1028, and COG0583, showed a significantly higher abundance (*p* < 0.01) in the full captivity breeding model compared to the other two breeding models, based on statistical analysis. Besides, the abundance of COG1595, COG0534, and COG4974 were significantly lower in the full captivity breeding model compared to the semi-captivity breeding model.

In the KOs prediction analysis, a total of 6,532 KOs were identified, including K03088 (*rpoE*; RNA polymerase sigma-70 factor, ECF subfamily), K02004 (putative ABC transport system permease protein), K00059 (*fabG*; 3-oxoacyl-[acyl-carrier protein] reductase [EC:1.1.1.100]), K02015 (iron complex transport system permease protein), K06180 (*rluD*; 23S rRNA pseudouridine1911/1915/1917 synthase [EC:5.4.99.23]), K03657 (*uvrD*, *pcrA*; DNA helicase II/ATP-dependent DNA helicase *PcrA* [EC:3.6.4.12]), K02016 (iron complex transport system substrate-binding protein), K03497 (*parB*, *spo0J*; chromosome partitioning protein, *ParB* family), K00615 (*tktA*, *tktB*; transketolase [EC:2.2.1.1]), and K02013 (iron complex transport system ATP-binding protein [EC:3.6.3.34]). The detailed abundance of each KO in each sample is presented in Supplementary Figure S3.

**Figure 10:**
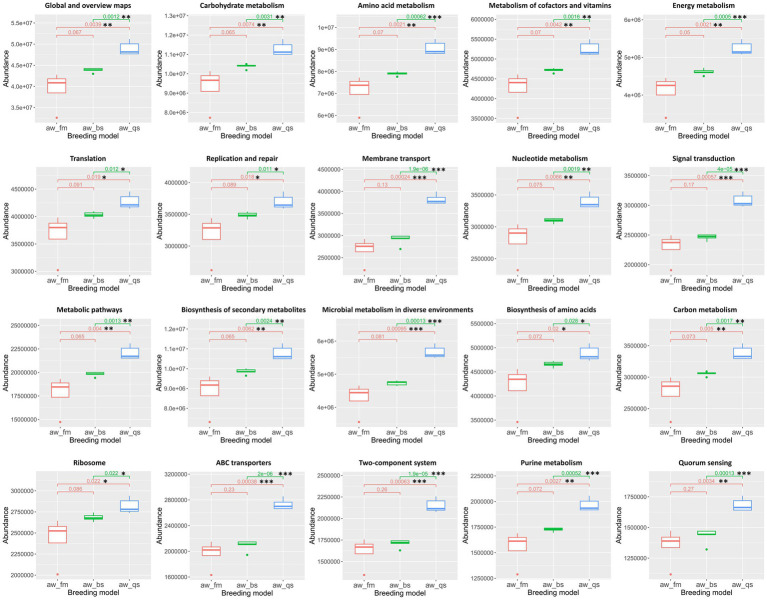
Boxplot representation of KEGG functional pathway abundances across different breeding models. The Kruskal-Wallis algorithm was employed to statistically analyze the differences in the predicted KEGG results among these models. The boxplots illustrate the distribution of abundance values for each pathway, with significant differences among the breeding models denoted. aw_fm, aw_bs, and aw_qs represents pure grazing, semi-captivity, and full captivity breeding models, respectively. “*” represents *p* < 0.05, “**” represents *p* < 0.01, and “***” represents *p* < 0.001.

To present a more detailed construction of microbiota functional predictions, we primarily analyzed the top 10 KEGG functional pathways with the highest abundance and their differences at the second and third levels of the KEGG pathway hierarchy. Additionally, we utilized the Kruskal-Wallis algorithm to statistically analyze the differences in predicted KEGG results among multiple breeding models (Figure 10).

About the top 10 enriched functional pathways, including Global and overview maps, Carbohydrate metabolism, Amino acid metabolism, Metabolism of cofactors and vitamins, Energy metabolism, Translation, Replication and repair, Membrane transport, Nucleotide metabolism, and Signal transduction, there were no significant differences in abundance between the pure grazing and semi-captivity breeding models at the second level of the KEGG pathway hierarchy. However, in the full captivity breeding model, these enriched functional pathways exhibited significantly higher abundances compared to the other two breeding models. Furthermore, an apparent gradual increase in abundance could be observed from the pure grazing breeding model to the semi-captivity breeding model, then to the full captivity breeding model.

Consistent with previous classification at the second level of the KEGG pathway hierarchy, the top 10 enriched functional pathways, including Metabolic pathways, Biosynthesis of secondary metabolites, Microbial metabolism in diverse environments, Biosynthesis of amino acids, Carbon metabolism, Ribosome, ABC transporters, Two-component system, Purine metabolism, and Quorum sensing, exhibited a comparable trend of gradual increase in abundance at the third level of the KEGG pathway hierarchy. These 10 pathways, at the third level of the KEGG pathway hierarchy, were significantly more abundant in the full captivity breeding model than the other two breeding models.

## 4 Discussion

In our sequencing analysis, we obtained approximately 80,184 clean reads for each sample, with an average Q30 rate of 91.42%. This high Q30 rate indicates a high quality of the sequencing data, suggesting the presence of a diverse and rich microbial community in the samples. Based on the analysis of gut microbiota profiles in different breeding models, it is evident that the breeding model significantly impacts the composition and diversity of the gut microbiota of Awang sheep. The ANOSIM analysis further confirms these differences, underscoring the importance of breeding models in determining gut microbiota composition. In assessing the alpha diversity indices of the gut microbiota in Awang sheep, we found that the Shannon index was significantly higher in the semi-captivity group compared to the other groups. This suggests that the gut microbiota community in the semi-captivity group harbors a greater number of species with a relatively even distribution, reflecting a balanced representation of multiple species. In comparison to our study, Zhang et al. (2023) reported that rumen bacterial richness and diversity indices, including the ACE index, Chao1 index, Shannon index, and Simpson index, were significantly lower in the pen-fed group compared to the pasture-fed group of tan lambs. It has been reported that variations in feed due to different rearing conditions often occur, suggesting that dietary components are an important mediating factor contributing to these differences in bacterial diversity (Kivistik et al., 2023). In the present study, the pure grazing group of Awang sheep were allowed to free-range, and mainly intake alpine meadows (composed of perennial grasses), and the semi-captivity group was provided meadows, hay, and genkwa root, the full captivity group was provided concentrates consisting of Corn, wheat bran, and soybean meal. The sheep of the semi-captivity group have access to a wider variety of food sources than other groups. This diversity in feed types may explain the higher Shannon index observed in the gut microbiota of the semi-captivity group.

At present, the gut microbiota composition of Tibetan Awang sheep has not been fully characterized. In our sequencing analysis, Firmicutes, Bacteroidetes, and Proteobacteria emerged as the most predominant phyla in all fecal samples collected from Awang sheep. This finding aligns with numerous previous studies that have revealed Firmicutes and Bacteroidetes as the dominant phyla in the guts of various mammals, including sheep, albeit with varying relative abundances across different species and breeds (Wang et al., 2021). For instance, In Sun et al.’s research, Firmicutes, Bacteroidetes, and Proteobacteria were the most predominant phyla in the gut of blue sheep (*Pseudois nayaur*), comprising over 92% of the total microbial composition (Sun et al., 2019). Furthermore, the relative abundance of Firmicutes and Bacteroidetes in the rumen fluid of pasture-fed tan lambs exceeded 85%. Consistent with these researches on sheep gut microbiota, the proportions of Firmicutes and Bacteroidetes in the gut of Awang sheep also average over 90%. In the current research, we have identified a diverse array of bacterial genera prevalent in the gut microbiota of Awang sheep, including *Christensenellaceae_R-7_group*, *Romboutsia*, *Acinetobacter*, *Rikenellaceae_RC9_gut_group*, *Bacteroides*, *Clostridium*, *Alistipes*, *Treponema*, and *Ruminococcus*. Notably, most of these bacteria, including *Christensenellaceae_R-7_group*, *Rikenellaceae*_*RC9_gut_group*, *Ruminococcus*, *Treponema*, and *Alistipes*, have also been detected and shown to be abundant in other sheep species such as European Mouflon (*Ovis orientalis musimon*), Blue Sheep (*Pseudois nayaur*) and Chinese Tan lambs (Sun et al., 2019; Zhang et al., 2023). According to previous research, *Christensenellaceae_R-7_group*, *Rikenellaceae*_*RC9_gut_group*, *Ruminococcus*, and *Treponema* were regarded as fiber-degrading bacteria and positively correlated with the rumen degradability of nutrients (Gu et al., 2024; Zhang et al., 2022). In addition, *Romboutsia* and *Acinetobacter*, though prevalent in Awang sheep, were not similarly abundant in these other sheep species. Moreover, *Ruminococcaceae*_*UCG-005* and *Ruminococcaceae*_*UCG-010* were prevalent in other sheep species and even in Tibetan goats (Sun et al., 2019; Zhang K. et al., 2021). Despite being commonly found in the rumen microbiota, the abundance of *Ruminococcaceae_UCG-005* (Li et al., 2023) and *Ruminococcaceae_UCG-010* (Han et al., 2021) was notably low in the gut microbiota of Awang sheep, as detected in the present study.

On the other hand, *Bacteroides* were prevalent in the microbiota of Awang sheep, which has also been reported to be abundant in Tibetan goats (Zhang K. et al., 2021). Furthermore, Liu H. et al. (2022) also found a higher abundance of *Bacteroides* in Qinghai Tibetan sheep and pointed out that *Bacteroides* are associated with high protein and low fiber forage nutrition. Given the extreme living conditions in Tibet, mammals residing there face significant survival challenges. Adaptive evolution has resulted in beneficial changes in gut microbiota genes related to energy metabolism, enabling these animals to better adapt to the harsh environment. It is conceivable that *Bacteroides*, or other specific microorganisms, may play a key role in facilitating the adaptation of highland *Caprinae* to the plateau environment. Indeed, previous research has shown that a higher proportion of *Bacteroides* is particularly advantageous for ruminants living at high altitudes regarding gut microbiota-mediated energy harvest (Sun et al., 2019). According to Liu et al.’s research (Liu et al., 2020), the low genetic diversity of Awang sheep, with only one haplogroup present, raises concerns about their vulnerability to extinction risks and the fragility of their gut microbiota ecosystem. Despite the low genetic diversity of Awang sheep, like most ruminants, Awang sheep shared a majority of microbial units with high abundance and the ability to digest fiber. Even in the study conducted by Liu et al., it was mentioned that Tibetan yak and sheep, though belonging to different species and genera, did not exhibit significant differences in their gut microbial diversity (Liu H. et al., 2022), suggesting that host genetics may not be a decisive factor influencing gut microbiota composition (Martinson et al., 2017).

The predominance of Firmicutes in the pure grazing and semi-captivity models might be attributed to the high fiber content in the forage-based diet, as Firmicutes are known to play a crucial role in fiber degradation (Liu et al., 2024). Consistent with Zhang et al.’s research (Zhang et al., 2023), we also observed a significantly higher abundance of Proteobacteria in the full captivity group than the pasture group. This finding could suggest a shift towards more proteolytic and saccharolytic metabolisms in the full captivity model, where sheep are provided with a more concentrated diet. At the genus level, the prevalence of certain taxa, such as *Christensenellaceae*_*R-7_group*, *Romboutsia*, and *Ruminococcus* across all feeding modes underscores their fundamental importance in maintaining gut health and homeostasis. The *Christensenellaceae_R-7_group*, a member of the emerging *Christensenellaceae* family, has garnered attention for its associations with host health (Waters and Ley, 2019). Notably, studies have demonstrated a positive correlation between *Christensenellaceae* and protein catabolism, as well as intestinal metabolites derived from dietary animal proteins (Manor et al., 2018). In the context of ruminants, the *Christensenellaceae_R-7_group* has been shown to enhance rumen development and augment nutrient absorption and digestion (Couch et al., 2020), suggesting its significance as a potential key player in the gastrointestinal tract microbiota of these animals. *Romboutsia* could produce short-chain fatty acids (SCFAs) such as acetic acid, propionic acid, and butyric acid through the fermentation of dietary fiber, and these SCFAs provide energy for the host and contribute to maintaining the stability of the intestinal environment (Wang and Jia, 2016). *Ruminococcus*, a common inhabitant of the intestinal tract of ruminants (Pang et al., 2022; Thapa et al., 2023; Wu et al., 2023), plays a pivotal role in the degradation of cellulose and hemicellulose within the rumen (Ma et al., 2022; Yeoman et al., 2021). Specifically, it produces various cellulases and hemicellulases that facilitate the conversion of dietary fiber into nutrients essential for the animal’s digestion and carbohydrate metabolism (Pope et al., 2010). Additionally, *Ruminococcus* can ferment cellobiose or cellulose, producing butyric acid, an important energy source for ruminants (Henderson et al., 2015). Of particular interest is the gradual decrease in abundance of these three genera, including *Christensenellaceae*_*R-7_group*, *Romboutsia*, and *Ruminococcus*, from pure grazing to semi-captive conditions and then to full captivity. The significant differences in abundance observed among the breeding models indicate that these genera may respond differently to changes in diet and environment. Taking into account the dietary components within each breeding model, the gradual decline in abundance of these genera from pure grazing to full captivity, along with the mentioned increase in abundance of Proteobacteria, suggests that more restrictive and concentrated feeding conditions may hinder the growth and activities of these beneficial microorganisms. Some researchers proposed that consuming a small number of concentrates may aid in maintaining a high abundance of the *Ruminococcus* community, which possesses the ability to digest cellulose, thereby enabling better dietary adaptation. Moreover, Zhang K. et al. (2021) also pointed out that a reasonable intensive feeding strategy is needed to prevent any harm to the well-being of Tibetan goats, while modern production systems provide ruminants with a much higher ratio of monosaccharides and proteins to non-starch polysaccharides than traditional grazing. Notably, *Bacteroides* were found to be more abundant in semi-captivity breeding models. Prior research has demonstrated that a higher proportion of *Bacteroides* is particularly advantageous for ruminants residing at high altitudes concerning gut microbiota-mediated energy harvest (Sun et al., 2019). In our study, the semi-captivity group inhabited an altitude of 4,006 meters, exceeding that of the pure grazing group (3,944 meters) and the full captivity group (3,562 meters). The observed increase in *Bacteroides* abundance with increasing breeding location altitude indirectly uncovered its crucial role in mediating energy acquisition among ruminants at high altitudes. On the other hand, *Acinetobacter* showed significantly increased abundance in the full captivity breeding model. It has been reported that some species of *Acinetobacter* are opportunistic pathogens that can cause infections when the host’s immune system is compromised (Dijkshoorn et al., 2007). An abnormally higher abundance of *Acinetobacter* may lead to intestinal dysfunction and disrupt Awang sheep’s gut microbiota balance. Therefore, it could be speculated that the Awang sheep of the full captivity group were at risk of *Acinetobacter* infection. Nevertheless, the specific source of *Acinetobacter* infection still needs to be further verified by examining the microbiota in this breeding environment.

The correlation analysis revealed complex interrelationships among the prevalent bacteria, suggesting that the gut microbiota operates as a tightly-knit ecological network. The positive correlations observed among genera known to be involved in fiber degradation (e.g., *Ruminococcus* and *Fibrobacter*) and short-chain fatty acid production (e.g., *Christensenellaceae*_R-7_group) indicate potential synergistic interactions that may contribute to the overall health and productivity of the animals (Zhang K. et al., 2021). Conversely, the negative correlations observed between some genera, such as *Acinetobacter* and *Ruminococcus*, suggest antagonistic interactions that may shift in the microbiota composition and function. While the pure grazing and semi-captivity groups exhibited diverse correlations among the genera compared to the full captivity group, no universal pattern was observed across all groups. Further research is needed to understand the impact of these inter-genus associations on the intestinal microbiota ecology and adaption of Awang sheep.

Utilizing Redundancy Analysis (RDA) and Mantel-Test on our comprehensive dataset, we have uncovered intriguing correlations between various environmental factors and the distribution patterns of dominant microbial genera in fecal samples from Awang sheep. This analysis further corroborates and supplements our previous findings on the differences in intestinal genera. As a supplement of our PCoA results, the RDA results indirectly explain a substantial portion of the total variation in community composition, consistent with previous findings on Tibetan ruminants by Liu H. et al. (2022). Specifically, we observed significant associations between altitude and the abundance of *Bacteroides*, as well as other genera including *Rikenellaceae_RC9_gut_group*, *Ruminococcus*, *Christensenellaceae_R-7_group*, and *Romboutsia*. Notably, *Bacteroides* has been reported to be prevalent in the gut of high-altitude host, including donkey (Guo et al., 2023), lizard (*Sceloporus grammicus*) (Montoya-Ciriaco et al., 2020), and Tibetan population (*Homo sapiens*) (Li et al., 2022). Given its prevalence and recent research indicating its role in improving glucose homeostasis and beneficial metabolic alterations in high-altitude exposed hosts (Liu et al., 2023), it is plausible that *Bacteroides* and other microbial populations harbor adaptive mechanisms that enable Awang sheep to thrive in high-altitude environments. Meteorological factors like wetness exhibit positive correlations with certain microbial genera, including *Romboutsia*, *Christensenellaceae_R-7_group*, and *Ruminococcus*. However, the underlying mechanisms behind these associations remain undocumented and require further investigation. In addition to altitude and wetness, diet is an equally significant environmental factor affecting the dominant genera from our previous analysis. Studies have shown that the transition of sheep from natural grazing to indoor feeding, depending on the feeding strategies such as semi-grazing with supplementation or barn feeding, led to distinct rumen fermentation patterns, major changes in ingested nutrients especially fiber, water-soluble carbohydrates, and starch, and impacted ruminants’ feed intake and gut microbiota (Cui et al., 2023). Traditionally, Tibetan ruminants graze extensively in alpine meadows that are rich in nutrients such as neutral detergent fiber, acid detergent fiber, and crude protein (Cui et al., 2021; Guo et al., 2024). Our current study reveals a positive correlation between the meadow diet and the abundance of multiple microbial genera, including *Christensenellaceae_R-7_group*, *Ruminococcus*, and *Romboutsia*. These microbial genera are generally associated with fiber degradation and energy extraction, suggesting that the alpine meadows, through the provision of plant-derived nutrients, foster the abundance of these beneficial microbes in the guts of Awang sheep (Gu et al., 2024; Zhang et al., 2022). In our RDA plot, we also observed that uniquely in the full captivity breeding models, the distribution of samples showed a significant positive correlation with concentrates and a strong negative correlation with meadows. This suggests that the exclusive feeding of concentrates and the absence of meadows in the fully captive breeding model are the primary factors contributing to the decreased abundance of fiber-degrading genera in the gut microbiota of Awang sheep.

PICRUSt predictions of microbial gene function provide valuable insights into potential functional differences among the gut microbiota of Awang sheep reared under varying conditions. Observed shifts in the abundance of COGs and KOs linked to metabolic pathways, signal transduction, and transport systems suggest that the gut microbiota may adapt to dietary and environmental changes associated with different breeding models. An increase in COGs related to signal transduction and transcriptional regulation in the fully captive model indicates an enhanced need for regulatory mechanisms to maintain homeostasis under more stressful conditions. However, the results from PICRUSt are vastly different from our expectations. Theoretically, in the full captivity breeding model, considering the low microbial diversity index in this group., the functional abundance of gut microbiota in Awang sheep should be less than that of the other two groups, yet the results do not reflect this. Probably, PICRUSt2 and other amplicon-based analyses have limitations in predicting strain-specific functionality due to their reliance on 16S rRNA gene amplification and analysis of hypervariable regions (Douglas et al., 2020). Consequently, further investigations leveraging metagenomics or proteomics are warranted to comprehensively understand the gut microbial flora’s function in Awang sheep.

## 5 Conclusion

In summary, we characterized the bacterial composition and functional potential of Awang sheep and revealed its significant differences across the three different breeding models. The identified prevalent and differentially abundant taxa, and the correlations among them, provide valuable insights into the ecological and functional complexity of the gut microbiota in Awang sheep. Based on the overall Shannon index and microbiota structure evaluation, the semi-captive breeding model is the most suitable feeding method. According to the comparative analysis of the bacterial community, it was suggested that feeding a certain proportion of forage along with a relatively small amount of feed can improve the adaptability of the intestinal microbiota of Awang sheep in the fully captive breeding model. Upon conducting an RDA analysis, we have established significant correlations between various environmental factors, including diet and altitude, and the abundance of dominant microbial genera. Future studies should aim to further explore the potential implications of these findings for animal health, nutrition, and production, as well as the mechanisms underlying the observed microbiota shifts.

## Data Availability

The original contributions presented in the study are publicly available. This data can be found here: https://www.ncbi.nlm.nih.gov/sra, accession number PRJNA1107353.

## Footnotes

^1^ https://cutadapt.readthedocs.io/en/stable/

^2^ https://benjjneb.github.io/dada2/tutorial_1_8.html

^3^ https://docs.qiime2.org/2024.2/tutorials/feature-classifier/

^4^ https://github.com/picrust/picrust2

^5^ https://www.python.org

^6^ https://www.r-project.org/

^7^ https://huttenhower.sph.harvard.edu/graphlan

^8^ http://huttenhower.sph.harvard.edu/galaxy/root?tool_id=lefse_upload

^9^ http://canoco5.com
