# Supplementary material for: Dietary and environmental factors affecting the dynamics of the gut bacteria in Tibetan Awang sheep (Ovis aries) across divergent breeding models
Source: Front Microbiol. 2025 Feb 5;16:1502898. doi: 10.3389/fmicb.2025.1502898 (PMC11852841; doi:10.3389/fmicb.2025.1502898)
Supplement: Supplementary file 1 [file Data_Sheet_1.pdf]

Supplementary Table 1 Summary of 16s rRNA high-throughput sequencing data

Sample	Raw Reads	Filtered	Input passed filter	denoised	Merged	Input merged	Non chimeric	Input non-chimeri c	ASV counts	Total_ ASVs
aw_fm_01	80493	76981	95.64%	73341	53942	67.01%	47290	58.75%	966	4141
aw_fm_02	80979	77497	95.70%	75321	64479	79.62%	60534	74.75%	1052	4141
aw_fm_03	81772	75546	92.39%	73256	58277	71.27%	55041	67.31%	677	4141
aw_fm_04	79969	75206	94.04%	73184	61751	77.22%	57865	72.36%	974	4141
aw_fm_05	79653	75724	95.07%	73640	63183	79.32%	59227	74.36%	1019	4141
aw_bs_01	78730	75384	95.75%	72983	61866	78.58%	57831	73.45%	1186	4141
aw_bs_02	80119	74185	92.59%	71990	58012	72.41%	54403	67.90%	693	4141
aw_bs_03	80231	76417	95.25%	74243	63450	79.08%	59039	73.59%	1116	4141
aw_bs_04	78973	76057	96.31%	73591	62284	78.87%	57917	73.34%	1180	4141
aw_bs_05	80310	76195	94.88%	74081	63025	78.48%	58638	73.01%	1096	4141
aw_qs_01	79795	76454	95.81%	74383	64242	80.51%	61668	77.28%	977	4141
aw_qs_02	81240	76502	94.17%	74184	62450	76.87%	59522	73.27%	912	4141
aw_qs_03	80428	76106	94.63%	73726	62505	77.72%	60138	74.77%	997	4141
aw_qs_04	78102	74672	95.61%	72142	61442	78.67%	58964	75.50%	990	4141
aw_qs_05	81965	78567	95.85%	76153	66157	80.71%	63605	77.60%	1032	4141

Note: "Sample" represents the sample ID, "RawReads" indicates the total number of raw reads in the original data, "filtered" refers to the number of reads after filtering, "denoised" stands for the number of reads after denoising, "merged" represents the number of reads after quality filtering, "non_chimeric" indicates the number of reads after chimera removal, and "Total_ASVs" denotes the total number of ASVs (Amplicon Sequence Variants).

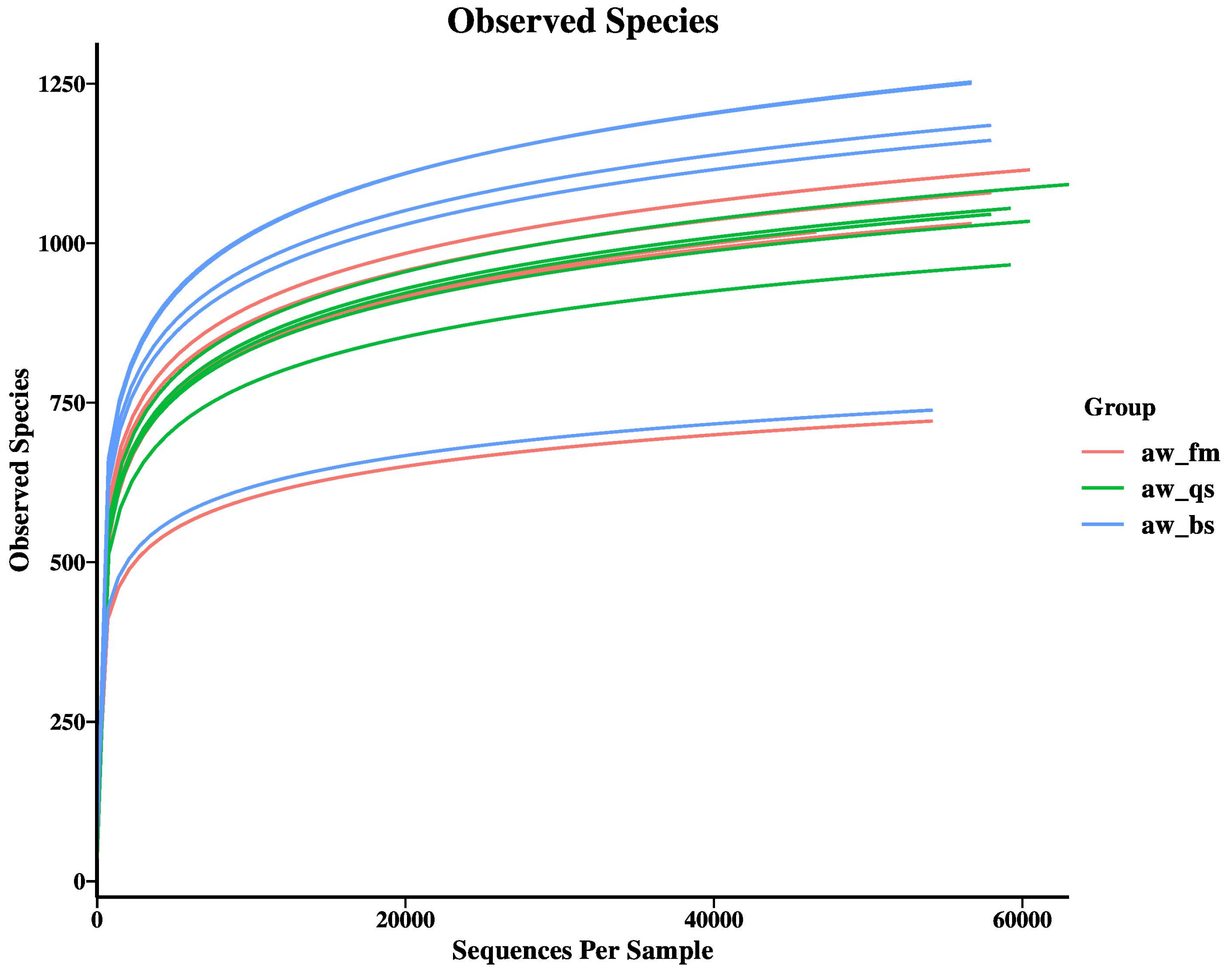

Supplementary Figure 1 Rarefaction curves

Note: aw_fm, aw_bs, and aw_qs represents pure grazing, semi-captivity, and full captivity breeding models, respectively.

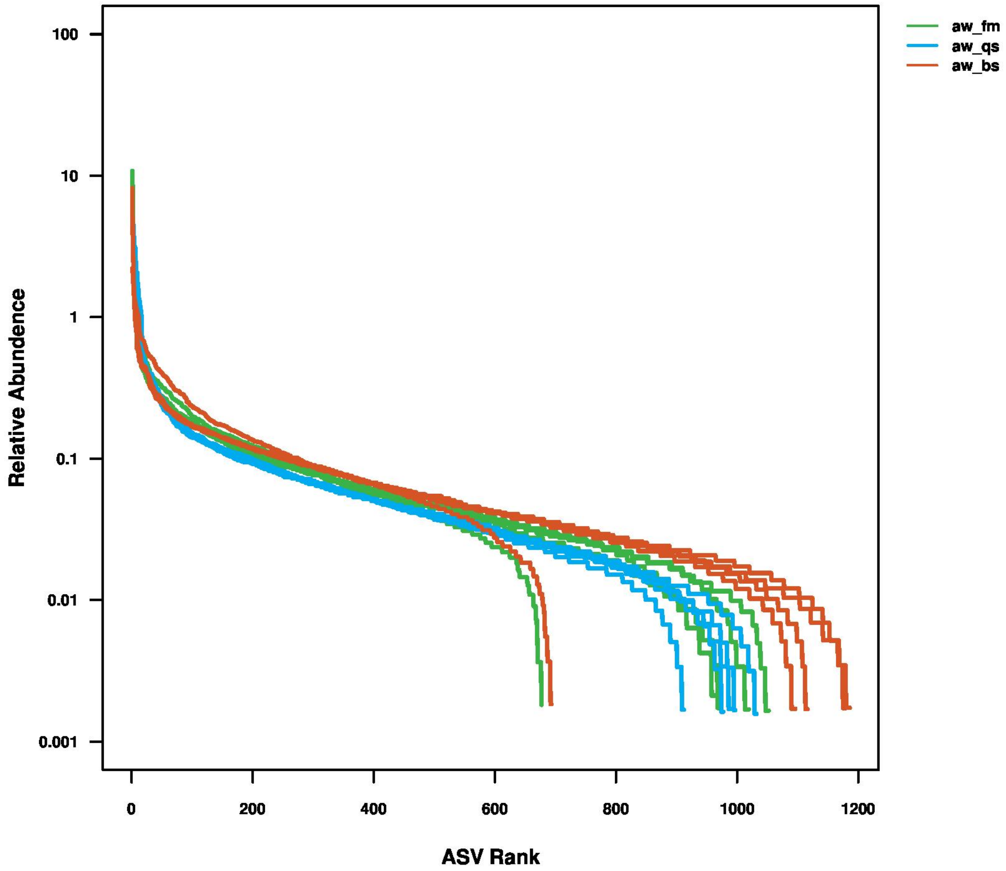

Supplementary Figure 2 Rank abundance curves

Note: aw_fm, aw_bs, and aw_qs represents pure grazing, semi-captivity, and full captivity breeding models, respectively.

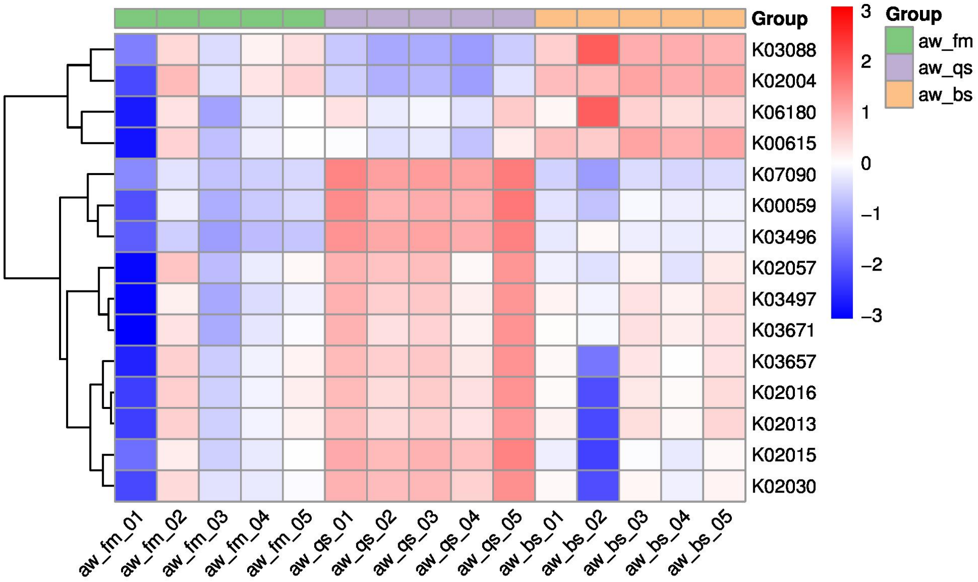

Supplementary Figure 3 The heatmap of the relative abundance of the top 15 KOs in each sample

Note: aw_fm, aw_bs, and aw_qs represents pure grazing, semi-captivity, and full captivity breeding models, respectively.
