# Supplementary material for: Dietary and environmental factors affecting the dynamics of the gut bacteria in Tibetan Awang sheep (Ovis aries) across divergent breeding models
Source: Front Microbiol. 2025 Feb 5;16:1502898. doi: 10.3389/fmicb.2025.1502898 (PMC11852841; doi:10.3389/fmicb.2025.1502898)
Supplement: Supplementary file 2 [file Image_1.pdf]

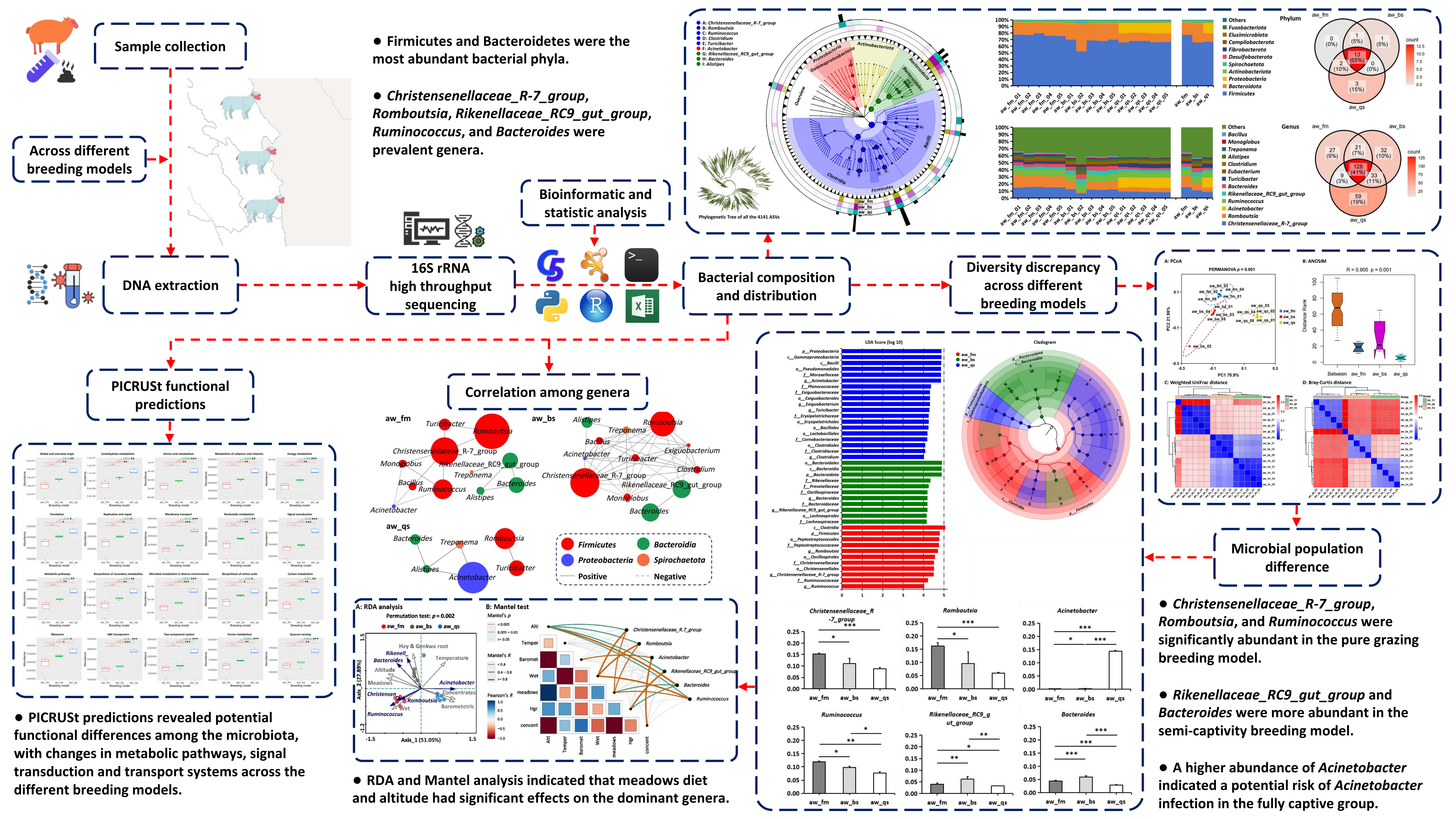

Sample collection
Across different breeding models
DNA extraction
16S rRNA high throughput sequencing
Bioinformatic and statistic analysis
Bacterial composition and distribution
Diversity discrepancy across different breeding models
Microbial population difference
PICRUSt functional predictions
Correlation among genera
● Firmicutes and Bacteroidetes were the most abundant bacterial phyla.
● Christensenellaceae_R-7_group, Romboutsia, Rikenellaceae_RC9_gut_group, Ruminococcus, and Bacteroides were prevalent genera.
Phylogenetic Tree of all the 4141 ASVs
Phylum
Genus
A: PCoA
B: ANOSIM
C: Weighted UniFrac distance
D: Bray-Curtis distance
LDA Score (log 10)
Cladogram
aw_fm
aw_bs
aw_qs
Firmicutes
Bacteroidia
Proteobacteria
Spirochaetota
Positive
Negative
A: RDA analysis
Permutation test: p = 0.002
B: Mantel test
● Christensenellaceae_R-7_group, Romboutsia, and Ruminococcus were significantly abundant in the pure grazing breeding model.
● Rikenellaceae_RC9_gut_group and Bacteroides were more abundant in the semi-captivity breeding model.
● A higher abundance of Acinetobacter indicated a potential risk of Acinetobacter infection in the fully captive group.
● PICRUSt predictions revealed potential functional differences among the microbiota, with changes in metabolic pathways, signal transduction and transport systems across the different breeding models.
● RDA and Mantel analysis indicated that meadows diet and altitude had significant effects on the dominant genera.
